# Correction: Díaz-Aljaro et al. Quantitative Microvascular Change Analysis Using a Semi-Automated Algorithm in Macula-on Rhegmatogenous Retinal Detachment Assessed by Swept-Source Optical Coherence Tomography Angiography. *Diagnostics* 2024, *14*, 750

**DOI:** 10.3390/diagnostics14111107

**Published:** 2024-05-27

**Authors:** Pablo Díaz-Aljaro, Xavier Valldeperas, Laura Broc-Iturralde, Nevena Romanic-Bubalo, Ignacio Díaz-Aljaro, Zhongdi Chu, Ruikang K. Wang, Javier Zarranz-Ventura

**Affiliations:** 1 Department of Ophthalmology, Hospital Universitari Germans Trias i Pujol, 08916 Badalona, Spain; pablodiazaljaro@gmail.com (P.D.-A.); laurabroc@gmail.com (L.B.-I.); nevenaromanic@gmail.com (N.R.-B.); 2 Department of Surgery, Universitat Autònoma de Barcelona (UAB), 08035 Barcelona, Spain; 3 Department of Ophthalmology, Hospital Clínico Universidad de Chile, Santiago 8380456, Chile; diazignacio3@gmail.com; 4 Department of Bioengineering, University of Washington, Seattle, WA 98195-5061, USA; zhongdichu@gmail.com (Z.C.); wangrk@uw.edu (R.K.W.); 5 Department of Ophthalmology, Hospital Clínic de Barcelona, 08036 Barcelona, Spain; jzarranz@hotmail.com

In the original publication [1], there was a mistake in Table 2 as published. VDI data for the deep capillary plexus was deleted by mistake. The corrected Table 2 appears below. The authors state that the scientific conclusions are unaffected. This correction was approved by the Academic Editor. The original publication has also been updated.

## Figures and Tables

**Table 2:** A comparison of the superficial capillary and deep capillary plexuses optical coherence tomography angiography (OCTA) parameters between the eyes with rhegmatogenous retinal detachment (RRD) and the fellow eyes.

	**Superficial Capillary Plexus (*n* = 23)**		
	**RRD**	**Fellow Eyes**	***p* ^a^**
VDI			0.0425
Mean ± SD	18.6 ± 1.1	19.2 ± 0.7	
Median (IqR)	18.8 (17.6–19.4)	19.3 (18.9–19.8)	
VAD			0.2380
Mean ± SD	0.43 ± 0.02	0.44 ± 0.01	
Median (IqR)	0.43 (0.42–0.44)	0.43 (0.43–0.44)	
VSD			0.3596
Mean ± SD	0.17 ± 0.01	0.16 ± 0.01	
Median (IqR)	0.17 (0.16–0.17)	0.16 (0.16–0.17)	
	**Deep Capillary Plexus (*n* = 23)**		
	**RRD**	**Fellow Eyes**	***p* ^a^**
VDI			0.0473
Mean ± SD	18.6 ± 0.6	19.0 ± 0.4	
Median (IqR)	18.6 (18.1–190.0)	19.0 (18.7–19.29)	
VAD			0.0425
Mean ± SD	0.38 ± 0.03	0.40 ± 0.02	
Median (IqR)	0.38 (0.36–0.41)	0.40 (0.39–0.42)	
VSD			0.1305
Mean ± SD	0.15 ± 0.01	0.15 ± 0.01	
Median (IqR)	0.15 (0.14–0.16)	0.15 (0.15–0.16)	

^a ^Mann–Whitney U-test. Study eyes—eyes with RRD; SD—standard deviation; IqR—interquartile range; VDI—vessel diameter index; VAD—vessel area density; VSD—vessel skeletal density.
