# Supplementary material for: Quantitative Microvascular Change Analysis Using a Semi-Automated Algorithm in Macula-on Rhegmatogenous Retinal Detachment Assessed by Swept-Source Optical Coherence Tomography Angiography
Source: Diagnostics (Basel). 2024 Apr 1;14(7):750. doi: 10.3390/diagnostics14070750 (PMC11011992; doi:10.3390/diagnostics14070750)
Supplement: Supplementary file 1 [file diagnostics-14-00750-s001.zip › diagnostics-2905422-supplementary.pdf]

Table S1. Spearman correlation coefficients of the optical coherence tomography angiography (OCTA) parameters between eyes with rhegmatogenous retinal detachment and their fellow eyes.

	**Superfical capillary plexus**					
	VDI RRD	VDI FE	VAD RRD	VAD FE	VSD RRD	VSD FE
VDI RRD SCC p	1	0.188<br>0.3910	-0.287<br>0.1850	-0.001<br>0.9964	-0.844<br><0.0001	-0.259<br>0.2329
VDI FE SCC p	0.188<br>0.3910	1	0.174<br>0.4274	0.153<br>0.4854	-0.005<br>0.9822	-0.711<br>0.0001
VAD RRD SCC p	-0.287<br>0.1850	0.174<br>0.4274	1	0.304<br>0.1580	0.678<br>0.0004	0.226<br>0.2992
VAD FE SCC p	-0.001<br>0.9964	0.153<br>0.4854	0.304<br>0.1580	1	0.215<br>0.3236	0.434<br>0.0386
VSD RRD SCC p	-0.844<br><0.0001	-0.005<br>0.9822	0.678<br>0.0004	0.215<br>0.3236	1	0.302<br>0.1608
VSD FE SCC p	-0.259<br>0.2329	-0.711<br>0.0001	0.226<br>0.2992	0.434<br>0.0386	0.302<br>0.1608	1
	**Deep capillary plexus**					
	VDI RRD	VDI FE	VAD RRD	VAD FE	VSD RRD	VSD FE
VDI RRD SCC p	1	0.250<br>0.2499	0.503<br>0.0144	0.113<br>0.6088	0.011<br>0.9607	0.061<br>0.7812
VDI FE SCC p	0.250<br>0.2499	1	-0.011<br>0.9607	-0.119<br>0.5900	-0.080<br>0.7166	-0.303<br>0.1594
VAD RRD SCC p	0.503<br>0.0144	-0.011<br>0.9607	1	0.403<br>0.0565	0.846<br><0.0001	0.357<br>0.0948
VAD FE SCC p	0.113<br>0.6088	-0.119<br>0.5900	0.403<br>0.0565	1	0.318<br>0.1390	0.966<br><0.0001
VSD RRD SCC p	0.011<br>0.9607	-0.080<br>0.7166	0.846<br><0.0001	0.318<br>0.1390	1	0.266<br>0.2202
VSD FE SCC p	0.910<br><0.0001	0.037<br>0.8685	0.601<br>0.0024	0.150<br>0.4939	0.144<br>0.5113	0.133<br>0.5440

SCC: Spearman correlation coefficient; RRD: Eye with rhegmatogenous retinal detachment; FE: Fellow eye; VDI: vessel diameter index; VAD: Vessel area density; VSD: Vessel skeleton density.
